# Geriatric Assessment of Older Patients Receiving Trabectedin in First-Line Treatment for Advanced Soft Tissue Sarcomas: The E-TRAB Study from The German Interdisciplinary Sarcoma Group (GISG-13)

**DOI:** 10.3390/cancers16030558

**Published:** 2024-01-28

**Authors:** Bernd Kasper, Daniel Pink, Christian Rothermundt, Stephan Richter, Marinela Augustin, Attila Kollar, Annegret Kunitz, Wolfgang Eisterer, Verena Gaidzik, Thomas Brodowicz, Gerlinde Egerer, Peter Reichardt, Peter Hohenberger, Markus K. Schuler

**Affiliations:** 1 Sarcoma Unit, Mannheim Cancer Center (MCC), Mannheim University Medical Center, University of Heidelberg, 68167 Mannheim, Germany; 2 Department of Oncology and Palliative Care, HELIOS Klinikum Bad Saarow, 15526 Bad Saarow, Germany; daniel.pink@helios-gesundheit.de; 3 Department of Internal Medicine C, University Medicine Greifswald, 17475 Greifswald, Germany; 4 Department of Medical Oncology and Hematology, Kantonsspital St. Gallen, 9007 St. Gallen, Switzerland; christian.rothermundt@kssg.ch; 5 Medical Department I, University Hospital Carl Gustav Carus, Dresden University of Technology, 01307 Dresden, Germany; 6 Department of Hematology and Oncology, Paracelsus Medical University, 90419 Nuremberg, Germany; marinela.augustin@klinikum-nuernberg.de; 7 Department of Medical Oncology, Bern University Hospital, University of Bern, 3010 Bern, Switzerland; attila.kollar@insel.ch; 8 Department of Hematology, Oncology and Palliative Medicine, Vivantes Klinikum Spandau, 13585 Berlin, Germany; annegret.kunitz@vivantes.de; 9 Clinical Division of Oncology, Department of Internal Medicine, Medical University Graz, 8036 Graz, Austria; wolfgang.eisterer@kabeg.at; 10 Clinic for Internal Medicine III, University Hospital Ulm, 89081 Ulm, Germany; verena.gaidzik@uniklinik-ulm.de; 11 Vienna General Hospital (AKH), Medizinische Universität Wien, 1090 Vienna, Austria; thomas.brodowicz@meduniwien.ac.at; 12 Department of Internal Medicine V, Heidelberg University Hospital, 69120 Heidelberg, Germany; gerlinde.egerer@med.uni-heidelberg.de; 13 Department of Oncology, Helios Klinikum Berlin-Buch, 13125 Berlin, Germany; peter.reichardt@helios-gesundheit.de; 14 Division of Surgical Oncology and Thoracic Surgery, University Medical Center Mannheim, Mannheim Medical Faculty, University of Heidelberg, 68167 Mannheim, Germany; 15 Onkologischer Schwerpunkt am Oskar-Helene Heim, 14195 Berlin, Germany

**Keywords:** geriatric assessment, patient-reported outcome, PRO, soft tissue sarcoma, STS, trabectedin

## Abstract

**Simple Summary:**

Older patients ≥ 55 years represent more than 50% of all sarcoma patients and have a worse prognosis compared to younger patients. Age alone should not be a reason to deprive patients of standard treatment with chemotherapy and surgery, provided they are functionally fit enough and willing to take the risk of adverse events. The E-TRAB study evaluated the feasibility and prognostic value of a comprehensive geriatric assessment representing different domains such as activities of daily living, co-morbidities and Patient-Reported Outcomes (PROs) for quality of life (QoL) and treatment tolerability. The study included 69 patients with soft tissue sarcoma (STS) between 55 and 88 years who were unsuited for treatment with anthracyclines and ifosfamide and received trabectedin as first-line therapy. Results show evidence that specific geriatric screening instruments could help predict or limit adverse treatment effects and, thereby, optimize treatment strategies in older STS patients.

**Abstract:**

E-TRAB was a non-interventional, prospective trial investigating the feasibility and predictive value of geriatric assessments (GA) in older STS patients treated with trabectedin as first-line therapy. Primary endpoints were overall survival (OS), quality of life and individual clinical benefit assessed by the patient-reported outcome measures QLQ-C30 and PRO-CTCAE. Further, several GA tools were applied and correlated with clinical outcomes and treatment-related toxicities. The final analyses included 69 patients from 12 German-speaking sites. The median age of patients was 78 years (range: 55 to 88). Baseline data on PROs and GA identified a diverse population of older patients with respect to their global health status, although a large proportion of them suffered from limitations, required geriatric help and had a high risk of morbidity. The Cancer and Age Research Group (CARG) score classified 38%, 29% and 23% of the patients with low, intermediate and high risks for therapy-related side effects, respectively. Median OS was 11.2 months [95%CI: 5.6; 19.4]. The study confirmed that trabectedin as first-line treatment in older patients with STS has an acceptable and manageable safety profile. Potential prognostic factors for clinical outcome and therapy-related toxicity were identified among the GA tools. Long Timed Up and Go (TUG) showed a significant correlation to OS and early death, whereas a high CARG score (>9) was associated with an increase in unplanned hospitalizations and the incidence of toxicities grade ≥ 3.

## 1. Introduction

Soft tissue sarcomas (STS) are a heterogeneous group of tumors arising mainly from the embryonic mesoderm and can be localized anywhere in the body. They comprise more than 150 different histological tumor entities exhibiting great differences in terms of clinical behavior, pathogenesis and genetic alterations [1]. All over the world, especially in industrialized countries, the number of older people is constantly increasing. In the German Cancer Registry, almost 50% of sarcomas occur beyond the age of 65 years [2], with typical features such as a higher proportion of complex karyotypes and a higher rate of adverse prognostic factors [3].

If diagnosed at an early stage and complete surgical removal of all tumor manifestations can be achieved, the prognosis of STS is favorable [4]. However, in up to 50% of patients, distant metastases occur. In previous years, the median overall survival (OS) in the metastatic setting was reported to be 12 to 15 months [5,6]. More recently, median OS appears to have improved and ranged from 20 to 24 months in randomized trials [7].

With few effective targeted treatments available for most advanced and/or metastatic STS, doxorubicin and ifosfamide—which have been used for more than 30 years—still remain the backbone of systemic chemotherapy with well-known side effects such as neutropenia, thrombocytopenia and nephrotoxicity [8]. In most cases, patients with advanced STS have a poor prognosis, and the primary goal of treatment is disease control and palliation. Trabectedin [9] and pazopanib [10] were introduced beyond first-line therapy and significantly enriched the therapeutic armamentarium for patients with STS. Trabectedin (Yondelis^®^) is the first anticancer marine-derived drug, approved in the European Union in 2007 and currently in close to 80 other countries for the treatment of adults with advanced STS after the failure of anthracycline and ifosfamide or for those patients who are unsuited to receive these agents, with a comparatively improved toxicity profile.

Previous clinical trials conducted in Germany specifically studied the older STS patient population by evaluating pazopanib and trofosfamide in first-line treatment in comparison to standard single-agent therapy with doxorubicin [11,12]. There are isolated retrospective reports in the literature on the use of trabectedin in older patients [13,14]; however, this patient population has not been studied prospectively or in first-line treatment with trabectedin yet.

The use of adequate geriatric assessment tools and predictive tools for estimating the expected chemotherapy toxicity should help when deciding whether or not to use chemotherapy in older patients [15]. On the other hand, several clinical studies demonstrated that the integration of geriatric assessment-guided interventions significantly reduces the toxic effects of cancer treatments [16,17]. However, although geriatric assessment has been implemented as a standard of care in guidelines for treating older cancer patients [18], it is still rarely used in daily clinical practice. There are concerns among oncologists that geriatric assessment can only be implemented at great expense and does not add any value. In fact, many are not yet aware of validated geriatric assessment tools [19,20]. The collection of further evidence-based data obtained through clinical trials may improve the existing tools and raise awareness of their usefulness in the standard of care for older cancer patients and could help these tools find their way into clinical practice.

The German Interdisciplinary Sarcoma Group (GISG) predominantly evaluates questions with an interdisciplinary focus in the early stages of clinical testing (www.gisg.de (accessed on 29 December 2023)). GISG-13 (E-TRAB) focused on establishing a geriatric assessment in patients aged ≥55 years receiving trabectedin as first-line treatment for advanced and/or metastatic STS. In this study, a comprehensive geriatric assessment (GA) was carried out, including the following domains: Instrumental Activities of Daily Living (IADL), Mini Nutritional Assessment (MNA), Charlson Comorbidity Index (CCI), Geriatric Depression Scale (GDS), Timed up and Go (TUG). Additionally, predictive data of two independent geriatric screening instruments (G8 and the Cancer and Age Research Group [CARG] prediction tool) in regards to unexpected hospitalization, the occurrence of toxicities grade ≥ 3 and early death during the first six months of treatment were also implemented. Furthermore, an explorative analysis of Patient-Reported Outcomes (PRO) was performed using the 30-item core European Organization for the Research and Treatment of Cancer (EORTC) Quality of Live Questionnaire (EORTC QLQ-30) as well as the core version of the Patient-Reported Outcomes of the Common Terminology Criteria for Adverse Events (PRO-CTCAE) [21]. E-TRAB was a D.A.CH. project that recruited patients from Germany, Austria and Switzerland (ClinicalTrials.gov Identifier: NCT03022448).

## 2. Materials and Methods

E-TRAB was designed as a non-interventional, prospective, international, phase IV study to identify measures of feasibility and effectiveness for advanced and/or metastatic STS patients unsuited (for example, due to cardiac comorbidities) to receive standard first-line chemotherapy and/or ≥60 years and treated with trabectedin. To this end, PROs, GA and geriatric screening instruments, as well as records of disease progression and tolerability of therapy, were evaluated. The study was performed at 12 German-speaking study centers across Germany, Switzerland and Austria. According to the non-interventional nature of the study, all treatment decisions and diagnostic and therapeutic procedures followed the routine clinical practice and were at the discretion of the investigator.

The study was conducted in accordance with the ethical standards as laid down in the 1964 Declaration of Helsinki and its later amendments, guidelines for Good Clinical Practice and local regulations on clinical trials. The study was approved by each study site’s independent ethics committee. All patients signed an informed consent document prior to inclusion in the study.

Patients ≥ 60 years of age with a histologic diagnosis of advanced or metastatic STS of intermediate or high grade were eligible for the study. A limited number of patients < 60 years were allowed but had to be unsuited for treatment with anthracyclines and ifosfamide. All patients were suitable for first-line treatment with trabectedin according to the local Summary of Product characteristics (SmPC). Patients with contraindications for trabectedin, as outlined in the SmPC, were excluded.

**Figure 1:**
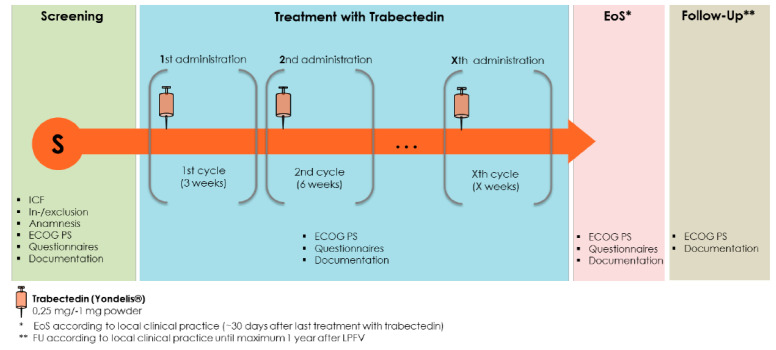
Scheme of study design. ICF—Informed consent; ECOG PS—Eastern Cooperative Oncology Group Performance Status; EoS—End of study.

The dosage and frequency of trabectedin treatment followed the specifications given in the SmPC and the standard medical care depending on the patient’s condition. The recommended dose of trabectedin for the treatment of STS is 1.5 mg/m^2^ body surface area administered as an intravenous infusion with a three-week interval between the cycles. Any dose modifications and changes in the dosing interval were in accordance with the SmPC and the investigator’s best clinical judgment. The number of treatment cycles was not restricted, and treatment was continued as long as the investigator felt there was benefit for the patient, even in the presence of apparent disease progression or until withdrawal of consent by the patient. After the end of treatment, follow-up assessments were scheduled at 3- to 6-month intervals. A summary of the study design is given in Figure 1.

The primary endpoints of the study were OS and QoL as measured by PROs. PROs as a subjective measure of clinical benefit were assessed using QLQ-C30 [22] and 18 items PRO-CTCAE [21]. After scoring according to the EORTC QLQ-C30 manual, all of the scales and single-item measures range in score from 0 to 100. A high score for the global health status/QoL represents a high QoL. A high score for a functional scale represents a high/healthy level of functioning, but a high score for a symptom scale/item represents a high level of symptomatology/problems.

As secondary endpoint, a self-administered GA was performed using several domains, including IADL [23], MNA [24], CCI [25], GDS [26], TUG [27]. Further, the predictive value of two different geriatric screening instruments (G8 [28], CARG prediction tool [29]) with regard to unplanned hospitalizations, occurrence of grade ≥ 3 toxicities and early death (within first six months after start of treatment) was assessed. The relationship between these assessments and disease-related endpoints such as OS, progression-free survival (PFS), overall response rate (ORR), disease control rate (DCR), time to onset of response, response duration and safety variables was examined.

OS was defined as the time between the start of trabectedin treatment and patient death from any cause, while PFS was defined as the time interval between the first administration of trabectedin and the date of disease progression or death, regardless of cause (whichever occurred first). Disease was regarded as progressed if either “disease progression” was recorded at the end of treatment, found by overall response assessment, or an adverse event (AE) occurred that indicated disease progression. Disease progression also included events of death. Patients without disease progression, being alive or considered lost to follow-up by the date of the database lock, were censored with the date of last contact. ORR was defined as the percentage of patients who achieved complete (CR) or partial response (PR) according to Response Evaluation Criteria in Solid Tumors (RECIST) version 1.1 [30], whereas DCR was defined as the percentage of patients with radiological CR, PR or stable disease (SD).

AEs were reported according to CTCAE version 4.0 and their relationship to trabectedin. Treatment-related AEs were followed until resolution or start of new therapy. AEs and serious AEs (SAEs) were recorded until 30 days after the last dose.

Statistical analyses were performed using the R software Version 4.1.1. Analyses were based on the intention-to-treat (ITT) population, which included all enrolled patients who provided informed consent and received at least one dose of trabectedin. All parameters were analyzed descriptively. Categorical variables were reported as absolute and relative frequencies, and continuous variables were described by number of observations, median and range (minimum to maximum). Frequency tables were provided for categorical variables and checked for dependencies by Fisher’s exact test. Approximate 95% confidence intervals (CI) were calculated for estimated rates (e.g., tumor control rate). Time-to-event endpoints (i.e., PFS and OS) were analyzed according to the Kaplan–Meier method and compared using the log-rank test. Cox regression for time-to-event outcomes and logistic regression for binary outcomes such as unplanned hospitalizations, occurrence of toxicities of grade ≥ 3 and early death were applied to assess the effects of the following parameters: age (<78 vs. ≥78 years), sex (male vs. female), ECOG PS (0 vs. 1 vs. 2), QLQ summary score (≤72.2 vs. >72.2), IADL (<1 vs. ≥1), MNA (normal vs. risk of malnutrition vs. malnutrition), CCI (≤2 vs. >2), GDS (normal vs. light to moderate), G8 score (≤14 vs. >14), CARG (low vs. intermediate vs. high), TUG (no vs. moderate restriction). For specifications of the different categories, please refer to the parameters listed in Table 1. Firstly, univariate regression was performed, including only one of these factors. Only factors with a significant effect in the univariate model at 10% significance level were considered in the multivariate model. The multivariate regression model was reduced by stepwise selection (mix of backward and forward selection) to relevant factors. For all analyses, *p*-value < 0.05 was considered statistically significant.

## 3. Results

### 3.1. Patient Disposition and Baseline Characteristics

**Figure 2:**
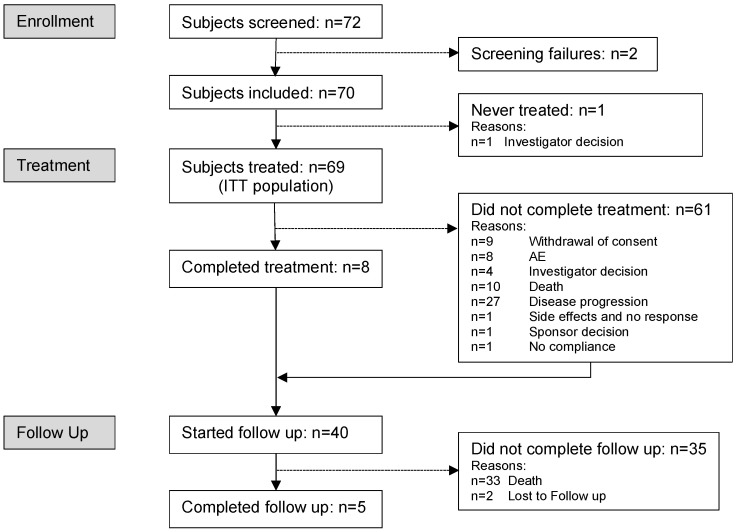
Consort chart.

Between 2 February 2017 and 31 December 2021, 70 patients were enrolled in 12 study centers in Austria, Germany and Switzerland, and 69 of them received at least one dose of trabectedin. All patients under trabectedin treatment were included in the ITT population (Figure 2).

Demographic and baseline characteristics are summarized in Table 1. Sixty-nine patients included in the ITT had a median age of 78 years (range: 55 to 88 years) at the time of inclusion in the study. Moreover, 56 patients were ≥70 years and 54 patients were >70 years; only two patients were younger than 60 years. Overall, 43.5% of patients were female and 56.5% were male. Twenty-two of the patients had an ECOG PS of 0, 38 had an ECOG PS of 1 and 9 patients had an ECOG PS of 2. The baseline data of PROs and GAs identified a diverse population of older patients with respect to their global health status, although a large proportion of them suffered from restrictions, required geriatric help and had a high risk of morbidity. Nonetheless, according to the CARG score, the risk for side effects was assessed as low or intermediate for 38% and 29%, respectively, of the population included in the study.

### 3.2. Extent of Exposure

Overall, the mean time on treatment with trabectedin was 3.6 months (range: 0 to 22.7 months). Study participants received a median of three cycles, with 24 (34.8%) patients receiving ≥6 cycles and up to a maximum of 21 cycles (2 patients, 2.9%). The median cumulative total dose was 4.20 mg/m^2^ (range: 0.94 to 30.0 mg/m^2^). Dose reductions were reported for 29 (42.0%) patients with a median of two (range: 1 to 10) cycles before the first reduction. Treatment interruptions were reported for 10 (14.5%) patients with a median of two (range: 1 to 5) cycles before the first interruption. Sixty-one (88.4%) patients discontinued treatment with trabectedin for the following reasons: disease progression (*n* = 27, 44.3%), death (*n* = 10, 16.4%), withdrawal of consent (*n* = 9, 14.8%), side effects (*n* = 9, 14.8%), investigator´s or sponsor´s decision (*n* = 5, 8.2%) and non-compliance (*n* = 1, 1.6%).

### 3.3. Primary Endpoints QLQ-C30 and PRO-CTCAE

**Figure 3:**
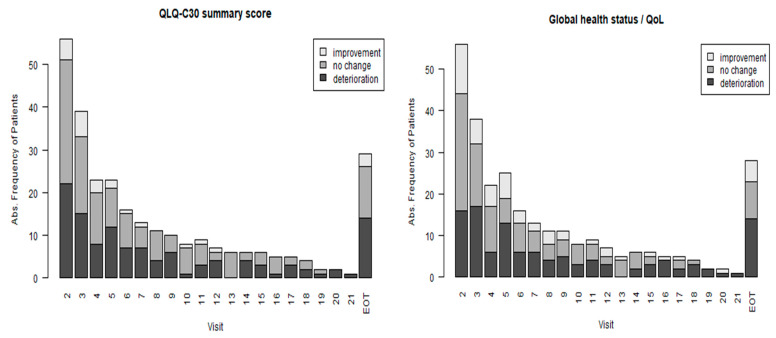
Changes in QLQ-C30 sum score and global health status over the treatment period. A change in score of >10 is regarded as clinically relevant deterioration or improvement. Please note that EOT is not an exactly defined time point and differs in time from patient to patient.

**Figure 4:**
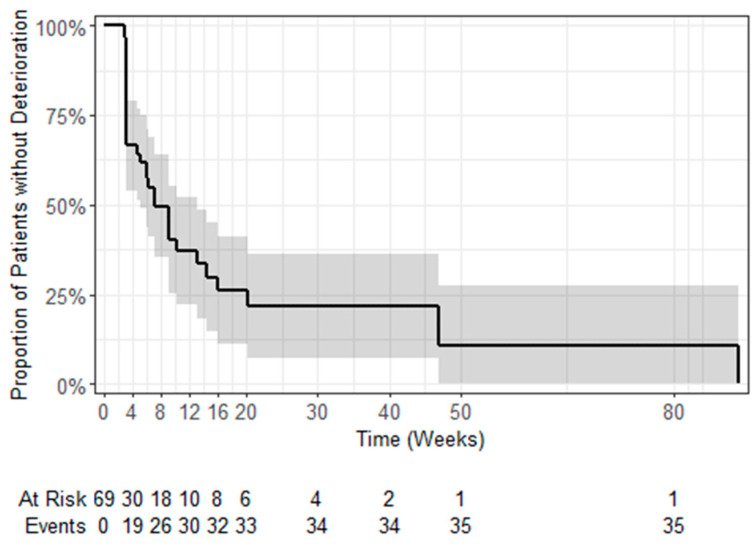
QLQ-C30 sum score: median time to deterioration, Kaplan–Meier estimates and 95% confidence intervals.

Overall, 67 (97.1%) patients provided valid answers to the QLQ-C30 questionnaire at the baseline visit (start of treatment if available, screening otherwise) and 29 (42.0%) at the end of treatment (EOT). Figure 3 illustrates the changes in QLQ-C30 sum score and global health status during treatment with trabectedin. Corresponding figures for the individual functional and symptom scales/items are provided in the supplementary materials (Figures S1 and S2). A change in score of >10 was regarded as clinically relevant deterioration or improvement. Among the patients with valid entries at baseline and EOT, three (4.3% of the ITT population) and five (7.2%) patients reported an improvement with regard to the QLQ-C30 sum score and global health status, respectively. No change was reported by 12 (17.4%) and 9 (13%) patients, respectively, whereas 14 (20.3%) patients felt that their health status had deteriorated. Overall, the median time to deterioration varied between 3.2 and 18 weeks for the different QLQ-C30 scales (see Figure 4 and Table 2).

**Figure 5:**
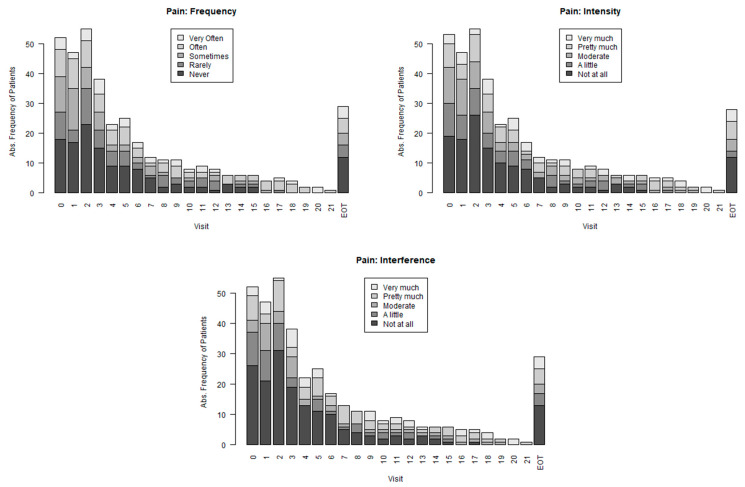
PRO-CTCAE: Change in pain frequency, intensity and interference with daily life during treatment with trabectedin. Please note that EOT is not an exactly defined time point and differs in time from patient to patient.

The PRO-CTCAE questionnaire provides a more differentiated record of symptoms since it covers different parameters such as intensity, frequency and interference with daily life (see in Figure 5 the extracted data for pain. Further Bar plots by question are provided in the supplementary materials (Figure S3).

The questionnaire was completed by 66 (95.7%) patients at baseline and by 29 (42.0%) patients at EOT.

### 3.4. Clinical Outcomes

**Figure 6:**
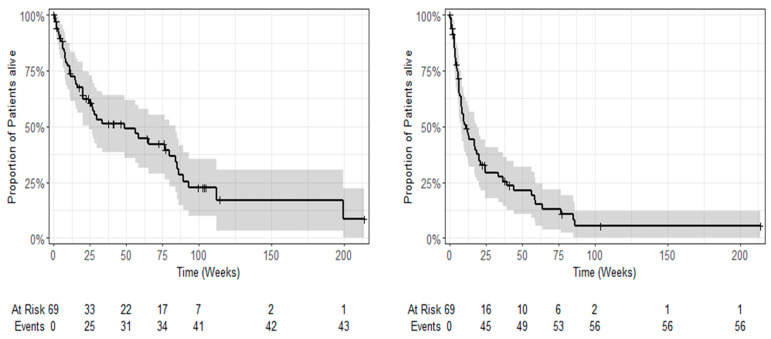
Kaplan–Meier plots of overall survival (**left**) and progression-free survival (**right**) (given are survival probabilities ± 95% confidence interval).

The median OS was estimated at 11.2 months [95%CI: 5.6; 19.4] (Figure 6). In total, 56 events of disease progression, including cases of death, were observed until the end of follow-up. The median PFS was estimated at 2.5 months [95%CI: 1.8; 4.3] (Figure 6).

Univariate Cox regression of OS with different factors revealed that the hazard ratios (HR) of MNA malnutrition and TUG moderate restriction were significantly different from 1 at the 10% level. However, in the multivariate model none of the possible predictors had an HR significantly different from 1 at the 5% level of significance (Table 3). Univariate Cox regression analysis for PFS revealed no significant HR for any of the predictors.

None of the patients achieved overall CR or PR at any visit. Thus, the ORR is 0 for all visits. The overall DCR (either CR, PR or SD) was 23.2% [95% CI: 0.130; 0.334]. Univariate logistic regression analysis showed no significant effect on DCR for any of the parameters tested.

The median time to onset of response was estimated at 6.92 weeks [95% CI: 3.99; 8.99]. The univariate Cox regression model showed evidence that the chance for disease control (i.e., reaching CR, PR or SD) is higher in the group with QLQ-C30 sum score of ≥72.2 than in the group with a sum score of <72.2 (Table 3). This corresponds to an earlier onset of response. The median time to onset of response for the groups with a sum score of <72.2 and ≥72.2 was 8.99 weeks [95% CI: 6.99; 10.13] and 4.13 weeks [95% CI: 3.85; 6.85], respectively. However, these data should be interpreted with caution as tumor assessment was performed as per investigators’ discretion and not at pre-defined time points throughout the study.

The median duration of response (patients with at least SD) was estimated at 27.3 weeks [95% CI: 9.43; 56]. In the multivariate model, none of the HRs was significantly different from 1. In the single Cox regression models, both MNA risk of malnutrition and a high-risk CARG sore had a significant effect on the duration of response. Here, the median duration of response was significantly shorter than in the other categories. However, due to the low number of patients, the results have to be interpreted with caution.

### 3.5. Predictive Value of G8 and CARG

The correlation between G8 and CARG sum scores was weak (Spearman correlation = 0.196), indicating that G8 and CARG predict different aspects of disease development. Regarding unplanned hospital admissions, the OR of a high CARG score vs. low CARG score is significantly different from 1, i.e., the odds of experiencing an unplanned hospitalization were five times higher for patients with a high CARG score compared to those with a low CARG score. All other variables investigated do not appear to be predictive of the occurrence of unplanned hospitalizations (Table 4).

Regarding the occurrence of grade ≥ 3 toxicities, the OR for G8 was not significantly different from 1, indicating that these factors do not have a significant effect. The OR for a high CARG score (>9) was 7.941, indicating a clear trend although not reaching statistical significance. The odds for the occurrence of a toxicity of at least grade 3 were 3.77 times higher in the group of patients with ECOG PS Grade 1 than in the subgroup of patients with ECOG PS Grade 0. All other variables investigated were not predictive of the occurrence of toxicities grade ≥ 3 (Table 4). It has to be noted that the overall number of toxicities grade ≥ 3 was low, which may reduce the power to reveal significant relationships.

A moderate restriction in TUG significantly increases the chance of an early death. The odds for an early death were 5.75 times higher for patients with a moderate restriction than for patients with no restriction in TUG. All other variables investigated do not appear to have an effect on early death (Table 4).

### 3.6. Safety

A total of 54 (78.3%) patients had at least one grade ≥ 3 AE. The most common (≥10% of patients) grade 3/4 AEs were decreased neutrophil count (*n* = 8, 11.6% of patients) and anemia (*n* = 7, 10.1% of patients). Eleven (15.9%) patients experienced grade 5 AEs, namely disease progression (*n* = 3), acute kidney injury (*n* = 1), infection (*n* = 1), liver injury (*n* = 1), pneumonia (*n* = 1), pulmonary embolism (*n* = 1), renal failure (*n* = 1), sepsis (*n* = 1) and vaginal hemorrhage (*n* = 1). For 47 (68.1%) patients, at least one SAE was reported.

A total of 55 (79.7%) patients had at least one trabectedin-related adverse drug reaction (ADR) of any grade, 35 (50.7%) of whom experienced grade ≥ 3 ADRs (Table 5). Sixteen (23.2%) patients had grade ≥ 3 ADRs leading to dose modifications, including drug withdrawal, and 22 (31.9%) of them experienced an SAE that was classified as trabectedin-related. No new toxicities or unexpected safety concerns were identified for trabectedin.

## 4. Discussion

There are only very few studies specifically focusing on the toxicity and activity of individual chemotherapy regimens given as first-line treatment to older STS patients. A prospective trial evaluating daily treatment with oral cyclophosphamide and prednisolone in patients aged ≥ 65 years unsuitable for conventional chemotherapy with doxorubicin and/or ifosfamide resulted in low toxicity and an ORR of 27% with a median PFS of 6.8 months [31]. A phase II study evaluated trabectedin as first-line treatment in patients with advanced STS unfit to receive standard chemotherapy for reasons such as stable arrhythmia, previous myocardial infarction and/or age ≥ 80 years [32].

In Germany, two first-line studies with a focus on systemic therapies in STS patients older than 60 years were completed [11,12]. These and other systemic first-line treatment studies are summarized in Table 6.

In regard to second- and later-line treatments of older patients with advanced STS, Le Cesne et al. conducted a retrospective pooled analysis of five phase II trials [13] and concluded that trabectedin is a feasible treatment in young and older patients with STS, comparable with that observed in the overall population, with an acceptable and manageable safety profile.

A subgroup analysis of 131 patients aged ≥65 years and enrolled in the phase III, randomized ET743-SAR-3007 study [35,37] of trabectedin vs. dacarbazine in patients with advanced liposarcoma or leiomyosarcoma also corroborated the previous observations showing that trabectedin not only significantly improved disease control over dacarbazine but also derived a similar benefit observed in younger patients [35]. In older patients, compared with dacarbazine, trabectedin significantly prolonged median PFS: 4.9 vs. 1.5 months (HR 0.40, *p* = 0.0002; overall population: 4.2 vs. 1.5 months, HR 0.55, *p* < 0.001) and showed a trend toward prolonged median OS (15.1 vs. 8.0 months; HR 0.72, *p* = 0.18; overall population: 13.7 vs. 13.1 months, HR 0.93, *p* = 0.49) [38].

It was hypothesized that for the patient population included in the herein-reported non-interventional E-TRAB study, the OS will not be beneath 10 months, and the QoL outcomes will be in the same range as for the standard therapy. In fact, a median PFS of 2.5 months and a median OS of 11.2 months are comparable with the populations of older patients receiving trabectedin in other studies (see Table 6 and [39]).

The E-TRAB trial investigated the feasibility and prognostic value of two geriatric screening instruments (G8 and CARG) and a comprehensive GA represented by different domains such as activities of daily living, co-morbidities and others (IADL, MNA, CCI, GDS, TUG), as well as PROs for QoL (QLQ-C30) and treatment tolerability (PRO-CTCAE). Results showed an overall good compliance of patients and oncologists to perform PROs and GAs. Regression analyses identified some of the parameters as potential prognostic factors for clinical outcomes and toxicity of therapy. Interestingly, age was not among these prognostic factors. Instead, there was evidence of impaired performance status or geriatric or functional status being of prognostic value. This has also been reported by Hamacher and colleagues investigating the role of geriatric variables in elderly patients with STS on toxicity and clinical outcomes in a post-hoc analysis of the EPAZ trial [40]. In this study, the prognostic factor of geriatric screening elements on PFS and OS was demonstrated, outlining the importance of geriatric screening and further investigation in clinical trials. In our study, univariate regression analysis revealed a significant correlation between moderate mobility restrictions, as identified by the TUG test, and OS, as well as early death. This is in line with data published by Soubeyran et al., who demonstrated that a long time in the TUG test is associated with a higher risk of early death [41].

The CARG score is a brief and validated tool to stratify patients into groups for low, medium, or high risk of severe chemotherapy toxicity. It includes five key geriatric assessment domains, selected laboratory values, age, tumor type and treatment intensity and was identified as a useful and feasible tool for supporting clinical decision-making [42,43]. In our study, a high CARG score (>9) was associated with a significant increase in unplanned hospitalizations and a trend for higher incidences of toxicities grade ≥ 3, while the G8 did not show this. Regarding the G8, this is in contrast to the findings of Lodewijckx and colleagues [44].

According to Chiusole and colleagues, CGA (comprehensive geriatric assessment) and oncological multidimensional prognostic index are prognostic values for the survival of patients with metastatic STS and are valuable tools to identify frail and high-risk patients who could benefit from an individualized oncogeriatric management approach to optimize treatment-related survival and reduce toxicity [45]. Further, results from the INTEGERATE study demonstrated that CGA led to better QoL and healthcare delivery in older patients receiving systemic anticancer treatment [36].

Data from our study using CGA in STS patients show evidence for a higher incidence of unplanned hospitalizations and toxicities grade ≥ 3 in patients with a high CARG score, indicating that patients at risk can be identified early and subjected to individualized therapy with reduced toxicity.

Although the study was limited by its non-interventional design that did not allow aligning time points for treatments and outcome assessments and finally included only a relatively small number of patients, it generated valuable data under real-life conditions in everyday clinical practice. Data confirmed that trabectedin as first-line treatment in older patients with STS has an acceptable and manageable safety profile. It must, however, be borne in mind that after 8 weeks of treatment, about 50% of the patients developed deterioration of their QoL. The study underlines the feasibility and important role of PROs and CGA as valuable tools for monitoring and even predicting treatment tolerability and efficacy, thus supporting individualized therapies that reconcile the side effects of chemotherapy and the QoL of older patients.

## 5. Conclusions

In conclusion, patients with STS should be treated in referral centers to ensure proper expertise with interdisciplinary treatment protocols. Older patients, who represent more than 50% of all sarcoma patients, have a worse prognosis compared to younger patients and are included only in a very small percentage of clinical trials. Whenever possible, they should be included in clinical studies, provided they are functionally fit enough and willing to take the risk of adverse events. Age alone should not be a reason to deprive patients of standard treatment with chemotherapy and surgery. Specific geriatric screening instruments, such as those implemented in the E-TRAB (GISG-13) trial, may help stratify patients to predict or limit adverse treatment effects and could be an important tool in optimizing treatment strategies for older patients with STS.

## Figures and Tables

**Table 1:** Patient demographics and baseline characteristics.

Parameter	ITT (*n* = 69)	
Age at study entry (years)	Median (range)	78 (55–88)
<60 years	*n* (%)	2 (2.9%)
≥60 years	*n* (%)	67 (97.1%)
Gender		
Female	*n* (%)	30 (43.5%)
Male	*n* (%)	39 (56.5%)
ECOG PS		
Grade 0	*n* (%)	22 (31.9%)
Grade 1	*n* (%)	38 (55.1%)
Grade 2	*n* (%)	9 (13.0%)
QLQ-C30 score ^1^		
Summary score	Median (range)	72.2 (22.3–99.5) (*n* = 67)
Global health status	Median (range)	58.3 (8.33–100) (*n* = 66)
Functional scales		
Physical functioning	Median (range)	66.7 (6.67–100) (*n* = 67)
Role functioning	Median (range)	66.7 (0–100) (*n* = 67)
Emotional functioning	Median (range)	66.7 (0–100) (*n* = 67)
Cognitive functioning	Median (range)	100 (16.7–100) (*n* = 67)
Social functioning	Median (range)	66.7 (0–100) (*n* = 67)
Symptom scales/items		
Fatigue	Median (range)	44.4 (0–100) (*n* = 67)
Nausea and vomiting	Median (range)	0 (0–100) (*n* = 67)
Pain	Median (range)	33.3 (0–100) (*n* = 67)
Dyspnea	Median (range)	33.3 (0–100) (*n* = 67)
Insomnia	Median (range)	33.3 (0–100) (*n* = 67)
Appetite loss	Median (range)	33.3 (0–100) (*n* = 67)
Constipation	Median (range)	33.3 (0–100) (*n* = 67)
Diarrhea	Median (range)	0 (0–100) (*n* = 67)
Financial difficulties	Median (range)	0 (0–100) (*n* = 67)
IADL total score	Median (range)	8 (2–8) (*n* = 68)
Patients with restrictions (<8)	*n* (%)	32 (46.4%)
Patients without restrictions (8)	*n* (%)	36 (52.2%)
MNA total score	Median (range)	27 (11–33) (*n* = 68)
Normal (≥24)	*n* (%)	55 (79.7%)
Risk of malnutrition (17–23.5)	*n* (%)	10 (14.5)
Malnutrition (<17)	*n* (%)	3 (4.3%)
CCI total score	Median (range)	8 (0–14) (*n* = 59)
Low mortality risk (≤2)	*n* (%)	12 (17.4%)
High mortality risk (>2)	*n* (%)	47 (68.1%)
GDS total score	Median (range)	3 (0–10) (*n* = 65)
Normal (0–5)	*n* (%)	59 (85.5%)
Light-to-moderate depression (6–10)	*n* (%)	6 (8.7%)
Severe depression (11–15)	*n* (%)	0
G8 total score	Median (range)	13 (5–16) (*n* = 68)
Geriatric care needed (≤14)	*n* (%)	50 (72.5%)
No geriatric care needed (>14)	*n* (%)	18 (26.1%)
TUG (Time in s)	Median (range)	8.0 (1–17) (*n* = 54)
Severe mobility restriction (≥30 s)	*n* (%)	0
Significant mobility restriction (20–29 s)	*n* (%)	0
Moderate mobility restriction (11–19 s)	*n* (%)	15 (21.7%)
No mobility restriction (<10 s)	*n* (%)	39 (56.5%)
CARG total score	Median (range)	7.0 (0–18) (*n* = 62)
Low risk for side effects (0–5)	*n* (%)	26 (37.7%)
Intermediate risk for side effects (6–9)	*n* (%)	20 (29.0%)
High risk for side effects (>9)	*n* (%)	16 (23.2%)

^1^ For QLQ-C30, all of the scales and single-item measures range in score from 0 to 100. A high score for the global health status/QoL represents a high QoL. A high score for a functional scale represents a high/healthy level of functioning, but a high score for a symptom scale/item represents a high level of symptomatology/problems.

**Table 2:** Individual QLQ-C30 scales: time to deterioration.

Scale	Number of Events	Median Time to Deterioration (Weeks) [95% CI]
QLQ-C30 sum score	36	7.14 [5.000; 12.994]
Global health status/QoL	35	7.14 [6.000; 12.857]
Physical functioning	38	5.86 [3.714; 9.000]
Role functioning	39	4.00 [3.143; 6.292]
Emotional functioning	29	9.00 [6.292; 22.000]
Cognitive functioning	33	6.86 [6.000; 9.143]
Social functioning	32	6.29 [3.149; 18.006]
Fatigue	46	3.15 [3.006; 5.000]
Nausea and vomiting	31	7.14 [4.286; 16.994]
Pain	28	13.00 [9.994; 20.137]
Dyspnea	23	14.29 [9.000; n.a.]
Insomnia	29	7.14 [6.994; 18.006]
Appetite loss	31	9.00 [6.006; 16.994]
Constipation	24	9.57 [6.994; n.a.]
Diarrhea	22	18.01 [10.006; n.a.]
Financial difficulties	15	n.a. [18.006; n.a.]

n.a. not applicable.

**Table 3:** Univariate logistic regression.

	OS	Time to Onset of Response	Duration of Response
Factor	HR [95% CI], *p*-Value	HR [95% CI], *p*-Value	HR [95% CI], *p*-Value
Age ≥ 78	1.130 [0.610; 2.095], 0.697	2.251 [0.788; 6.430], 0.130	0.700 [0.182; 2.691], 0.604
Sex (male)	1.169 [0.634; 2.155], 0.617	0.942 [0.339; 2.617], 0.909	1.068 [0.259; 4.401], 0.928
ECOG PS Grade 1	1.270 [0.634; 2.544], 0.501	0.951 [0.299; 3.028], 0.933	1.081 [0.205; 5.700], 0.927
ECOG PS Grade 2	1.664 [0.643; 4.309], 0.294	2.263 [0.409; 12.530], 0.350	0.230 [0.017; 3.114], 0.269
QLQ-C30 summary score > 72.2	0.698 [0.377; 1.292], 0.252	5.462 [1.325; 22.51], 0.019	2.192 [0.505; 9.517], 0.295
IADL at least one restriction	1.516 [0.831; 2.763], 0.175	1.241 [0.446; 3.452], 0.680	0.858 [0.244; 3.014], 0.811
MNA malnutrition	2.663 [0.627; 11.310], 0.185	1.226 [0.149; 10.097], 0.850	0.873 [0.099; 7.693], 0.902
MNA risk of malnutrition	2.288 [0.917; 5.714], 0.076	0.787 [0.215; 2.880], 0.717	6.232 [1.022; 37.996], 0.047
CCI > 2 (high mortality risk)	0.966 [0.403; 2.316], 0.937	1.113 [0.298; 4.152], 0.874	0.311 [0.056; 1.740], 0.184
GDS moderate	1.764 [0.688; 4.522], 0.237	2.966 [0.331; 26.61], 0.331	3.534 [0.366; 34.100], 0.275
G8 ≤ 14	1.025 [0.524; 2.005], 0.943	0.717 [0.252; 2.037], 0.532	1.907 [0.489; 7.446], 0.353
CARG intermediate (6–9)	1.298 [0.633; 2.661], 0.476	1.100 [0.328; 3.688], 0.878	0.217 [0.024; 1.992], 0.177
CARG high (>9)	0.752 [0.318; 1.778], 0.516	0.483 [0.119; 1.965], 0.310	10.12 [1.042; 98.337], 0.046
TUG moderate restriction	1.987 [0.971; 4.063], 0.060	0.602 [0.126; 2.865], 0.524	1.691 [0.188; 15.250], 0.639

OS—overall survival; HR—hazard ratio; CI—confidence interval.

**Table 4:** Univariate logistic regression.

	Unplanned Hospitalizations	Occurrence of Toxicities Grade ≥ 3	Early Death
Factor	OR [95% CI], *p*-Value	OR [95% CI], *p*-Value	OR [95% CI], *p*-Value
G8 > 14	1.105 [0.373; 3.273], 0.857	0.709 [0.173; 2.900], 0.632	0.867 [0.294; 2.559], 0.796
CARG intermediate (6–9)	0.955 [0.296; 3.078], 0.938	2.118 [0.543; 8.262], 0.280	1.048 [0.325; 3.378], 0.938
CARG high (>9)	5.056 [1.159; 22.060], 0.031	7.941 [0.898; 70.216], 0.062	0.857 [0.246; 2.983], 0.809
ECOG PS 1	0.714 [0.591; 4.970], 0.321	3.771 [1.048; 13.569], 0.042	1.533 [0.532; 4.422], 0.429
ECOG PS 2	0.800 0.168; 3.799], 0.779	2.000 [0.332; 12.046], 0.449	0.500 [0.099; 2.522], 0.401
IADL at least one restriction	1.491 [0.565; 3.933], 0.419	2.692 [0.751; 9.649], 0.128	2.083 [0.788; 5.506], 0.139
MNA risk of malnutrition	0.387 [0.033; 4.526], 0.449	2.512 [0.289; 21.8], 0.404	4.462 [0.868; 22.941], 0.073
MNA malnutrition	1.806 [0.422; 7.730], 0.425	0.558 [0.047; 6.693], 0.645	2.231 [0.191; 26.062], 0.522
TUG moderate restriction	2.783 [0.675; 11.48], 0.157	not estimable	5.750 [1.394; 23.716], 0.016

OR—odds ratio, CI—confidence interval.

**Table 5:** Treatment-related adverse drug reactions (ADRs) in at least ≥3% of patients and all grade-5 ADRs as reported by the investigators (all treated patients, *n* = 69) ^1^.

Treatment-Related ADR as Per NCI-CTC, Worst Case Per Patient (≥3% of Patients)	Grade 1 *n* = 28		Grade 2 *n* = 23		Grade 3 *n* = 31		Grade 4 *n* = 7		Grade 5 *n* = 2		Total *n* = 55	
	*n*	%	*n*	%	*n*	%	*n*	%	*n*	%	*n*	%
Nausea	14	20.3	5	7.2	5	7.2	-	-	-	-	24	34.8
Fatigue	13	18.8	5	7.2	-	-	-	-	-	-	18	26.1
Anemia	6	8.7	3	4.3	5	7.2	-	-	-	-	14	20.3
WBC decreased	5	7.2	4	5.8	2	2.9	3	4.3	-	-	14	20.3
ALAT increased	4	5.8	4	5.8	3	4.3	1	1.4	-	-	12	17.4
AST increased	9	13.0	-	-	2	2.9	-	-	-	-	11	15.9
Neutrophil count decreased	-	-	2	2.9	5	7.2	3	4.3	-	-	10	14.5
Vomiting	7	10.1	2	2.9	-	-	-	-	-	-	10 ^2^	14.5
Constipation	3	4.3	-	-	6	8.7	-	-	-	-	9	13.0
GGT increased	5	7.2	2	2.9	2	2.9	-	-	-	-	9	13.0
Platelet count decreased	5	7.2	1	1.4	-	-	2	2.9	-	-	8	11.6
Decreased appetite	6	8.7	1	1.4	-	-	-	-	-	-	7	10.1
Acute kidney injury	1	1.4	1	1.4	3	4.3	1	1.4	-	-	6	8.7
AP increased	3	4.3	-	-	3	4.3	-	-	-	-	6	8.7
Abdominal pain	3	4.3	1	1.4	1	1.4	-	-	-	-	5	7.2
Edema peripheral	4	5.8	1	1.4	-	-	-	-	-	-	5	7.2
Hypoalbuminemia	3	4.3	1	1.4	-	-	-	-	-	-	4	5.8
Hepatotoxicity	-	-	1	1.4	2	2.9	-	-	-	-	3	4.3
Liver disorder	-	-	1	1.4	2	2.9	-	-	-	-	3	4.3
Liver injury	-	-	-	-	-	-	-	-	1	1.4	1	1.4
Sepsis	-	-	-	-	-	-	-	-	1	1.4	1	1.4

^1^ The percentages relate to the number of patients in the Safety Analysis Set (all treated patients). ^2^ For one event of vomiting, the grade was not specified. NCI-CTC—National Cancer Institute Common Toxicity Criteria; ADR—adverse drug reaction; WBC—white blood cell count; ALAT—alanine aminotransferase; ASAT—aspartate aminotransferase; GGT—gamma-glutamyltransferase; AP—alkaline phosphatase.

**Table 6:** Summary of first-line studies in older patients.

Reference	Treatment	*n*	Age [Years]	ORR [%]	Median PFS [Months]	Median OS [Months]
E-TRAB study	Trabectedin	69	Median: 78	23.2 (DCR)	2.5	11.2
Younger et al. [33]	Doxorubicin-based chemotherapies	348	>65	14.9	3.5	10.8
Yousaf et al. [34]	Doxorubicin or others	120	>65	20	n.e.	6.5
Mir et al. [31]	Cyclophosphamide	27	≥65	27	6.8	n.e.
Grosso et al. [32]	Trabectedin	24	≥80	62 (DCR)	4	9
Hartmann et al. [12]	Trofosfamide	80	≥60	6.6	2.8	12.3
Grünwald et al. [11]	Pazopanib	81	≥60	n.e.	4.4	12.3
Jones et al. [35,36]	Trabectedin	94	>65	9	4.9	15.1

ORR—overall response rate; DCR—disease control rate; PFS—progression-free survival; OS—overall survival; n.e.—not evaluated.

## Data Availability

The data underlying this article will be shared upon reasonable request to the corresponding author.

## Supplementary Materials

The following supporting information can be downloaded at: https://www.mdpi.com/article/10.3390/cancers16030558/s1, Figure S1: Changes in QLQ-C30 functional scales over the treatment period; Figure S2: Changes in QLQ-C30 symptom scales/items over the treatment period; Figure S3: PRO-CTCAE Bar plots by question.
