# Supplementary material for: Geriatric Assessment of Older Patients Receiving Trabectedin in First-Line Treatment for Advanced Soft Tissue Sarcomas: The E-TRAB Study from The German Interdisciplinary Sarcoma Group (GISG-13)
Source: Cancers (Basel). 2024 Jan 28;16(3):558. doi: 10.3390/cancers16030558 (PMC10854510; doi:10.3390/cancers16030558)
Supplement: Supplementary file 1 [file cancers-16-00558-s001.zip › cancers-2825396-supplementary.pdf]

cancers

MDPI

Supplementary Materials

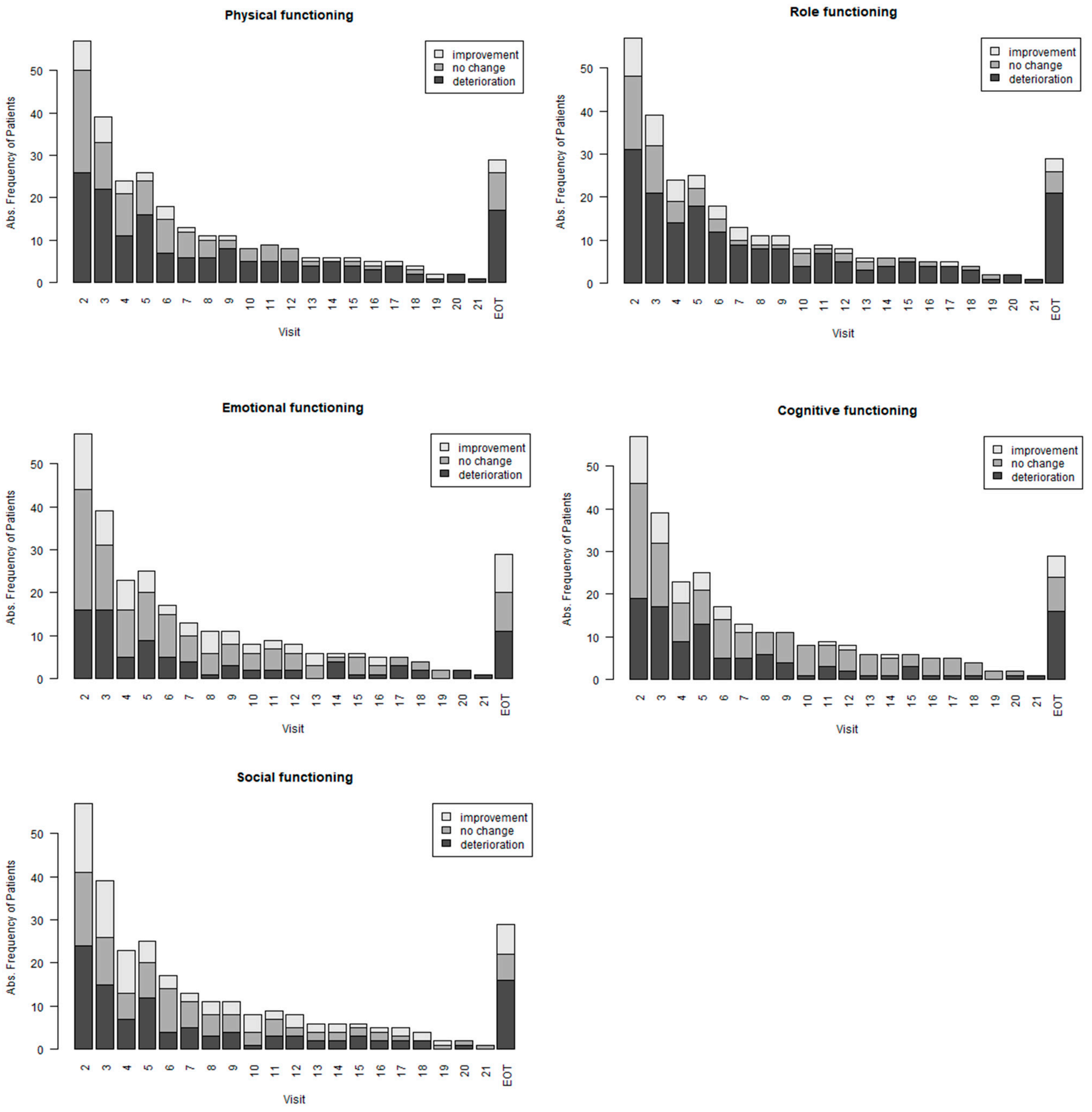

Figure S1. Changes in QLQ-C30 functional scales over the treatment period. A change in score of >10 is regarded as clinically relevant deterioration or improvement. Please note that the graph represents mean data for any visit where number of patients differs from visit to visit. Further, EOT is not an exactly defined time point and differs in time from patient to patient.

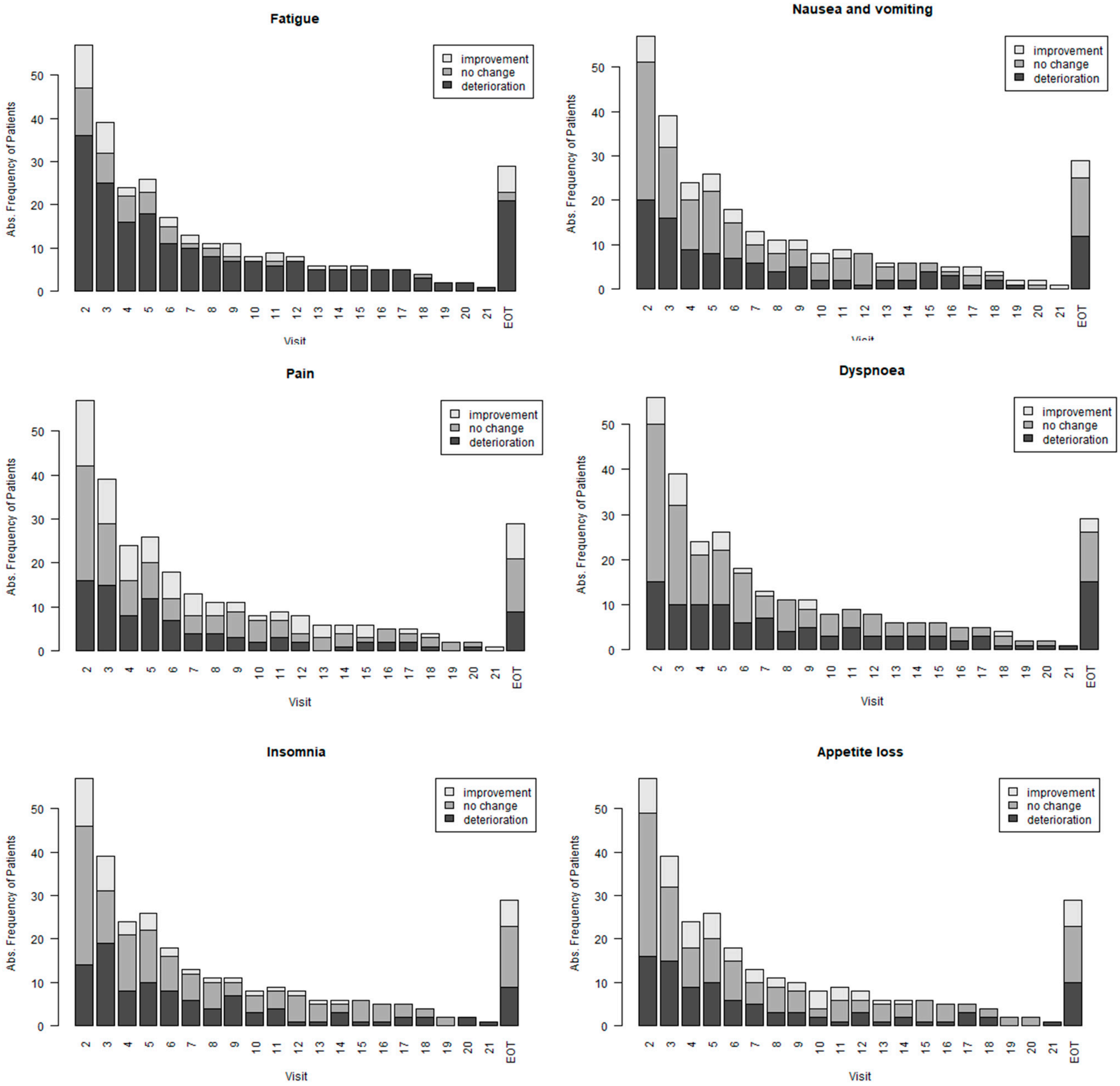
Fatigue
Nausea and vomiting
Pain
Dyspnoea
Insomnia
Appetite loss
improvement
no change
deterioration
Abs. Frequency of Patients
Visit
0
10
20
30
40
50
2
3
4
5
6
7
8
9
10
11
12
13
14
15
16
17
18
19
20
21
EOT

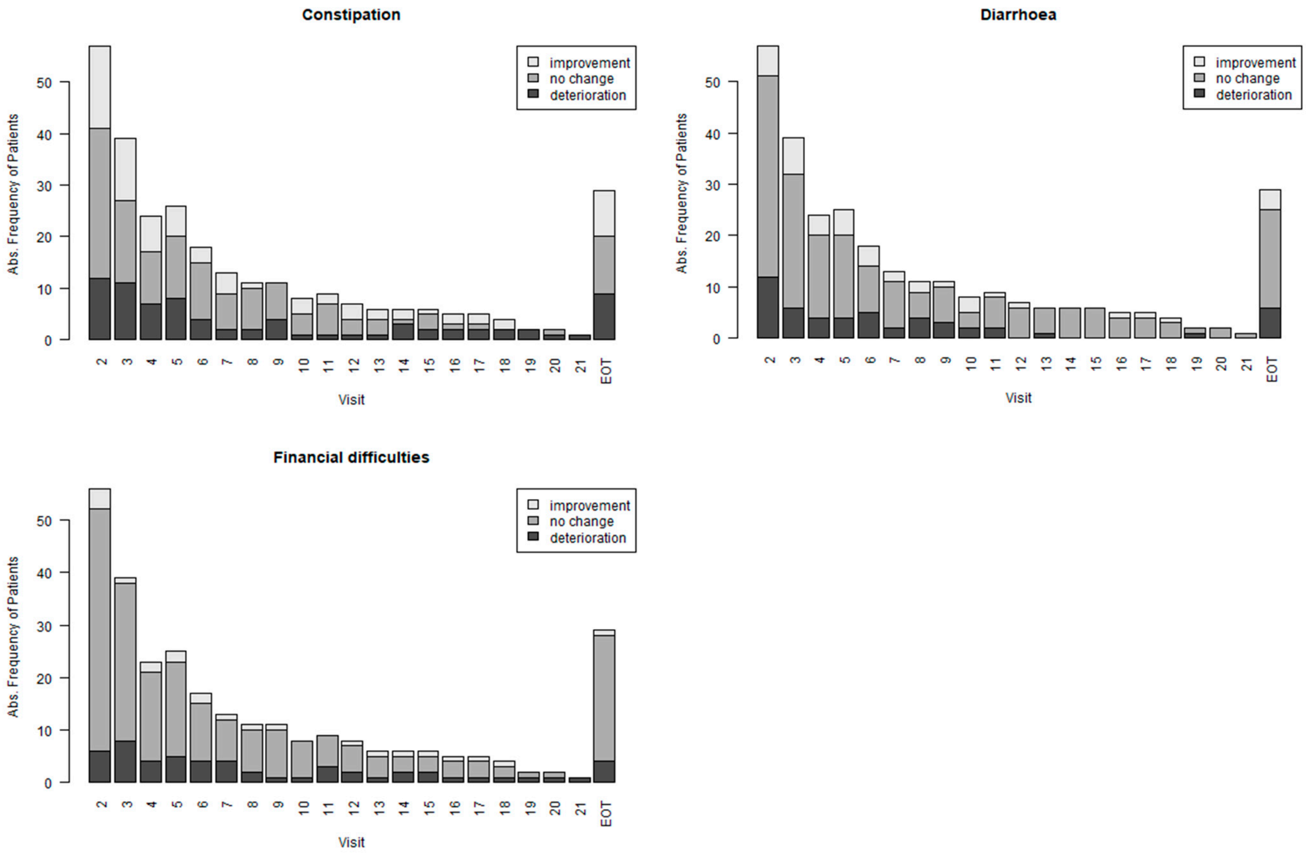

Figure S2. Changes in QLQ-C30 symptom scales/items over the treatment period. A change in score of >10 is regarded as clinically relevant deterioration or improvement. Please note that the graph represents mean data for any visit where number of patients differs from visit to visit. Further, EOT is not an exactly defined time point and differs in time from patient to patient.

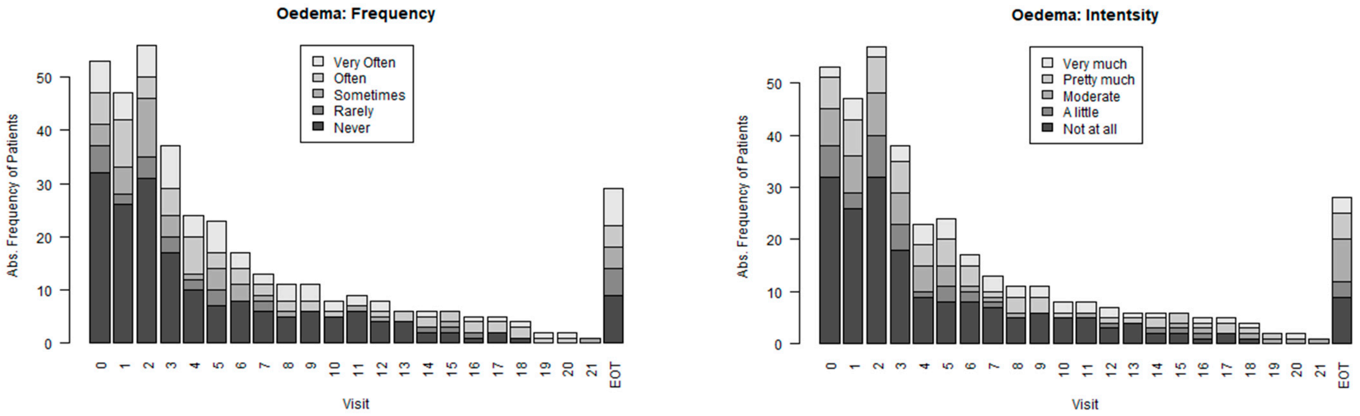

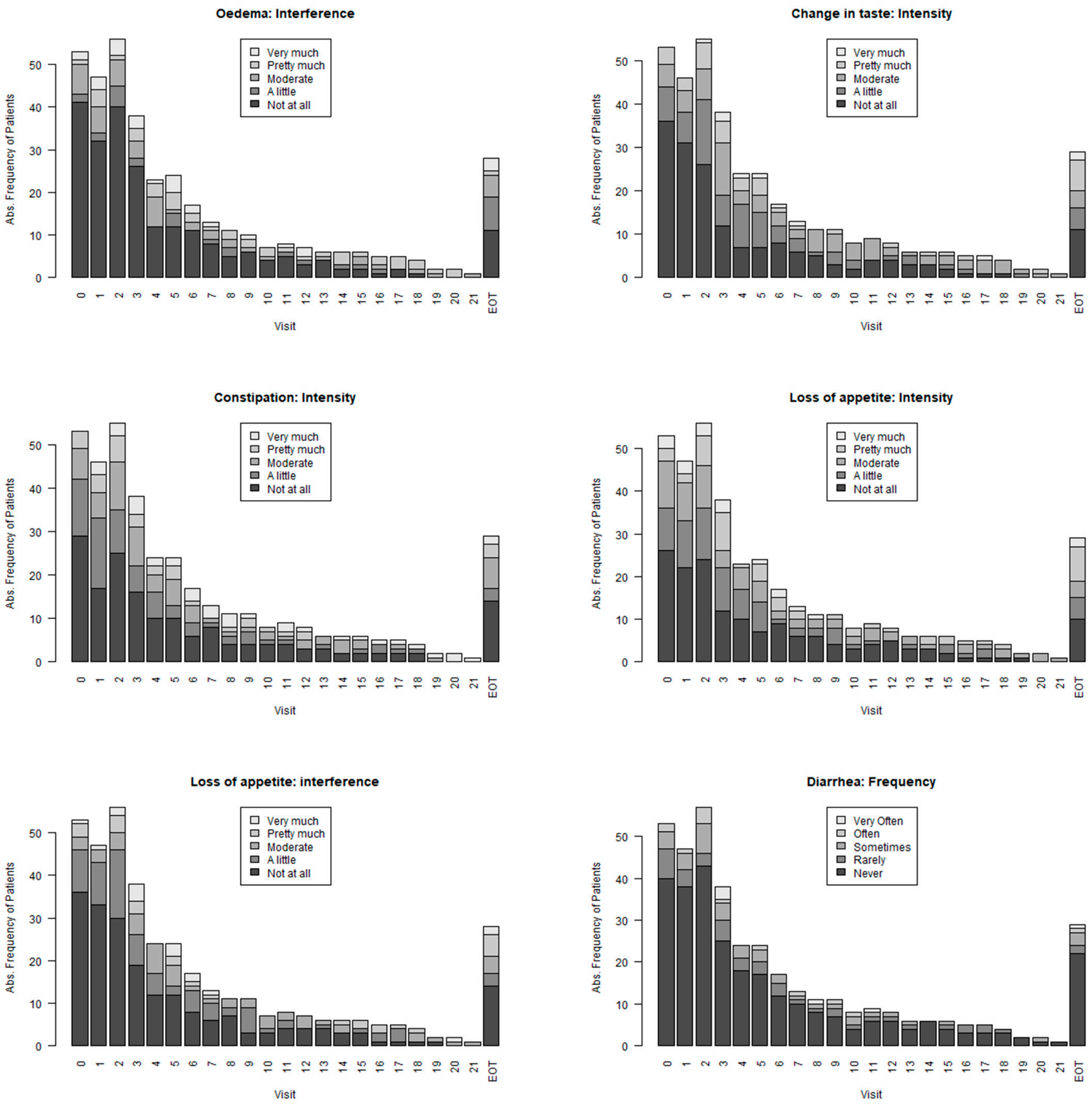
Oedema: Interference
Change in taste: Intensity
Constipation: Intensity
Loss of appetite: Intensity
Loss of appetite: interference
Diarrhea: Frequency
Very much
Pretty much
Moderate
A little
Not at all
Very Often
Often
Sometimes
Rarely
Never
Abs. Frequency of Patients
Visit
EOT

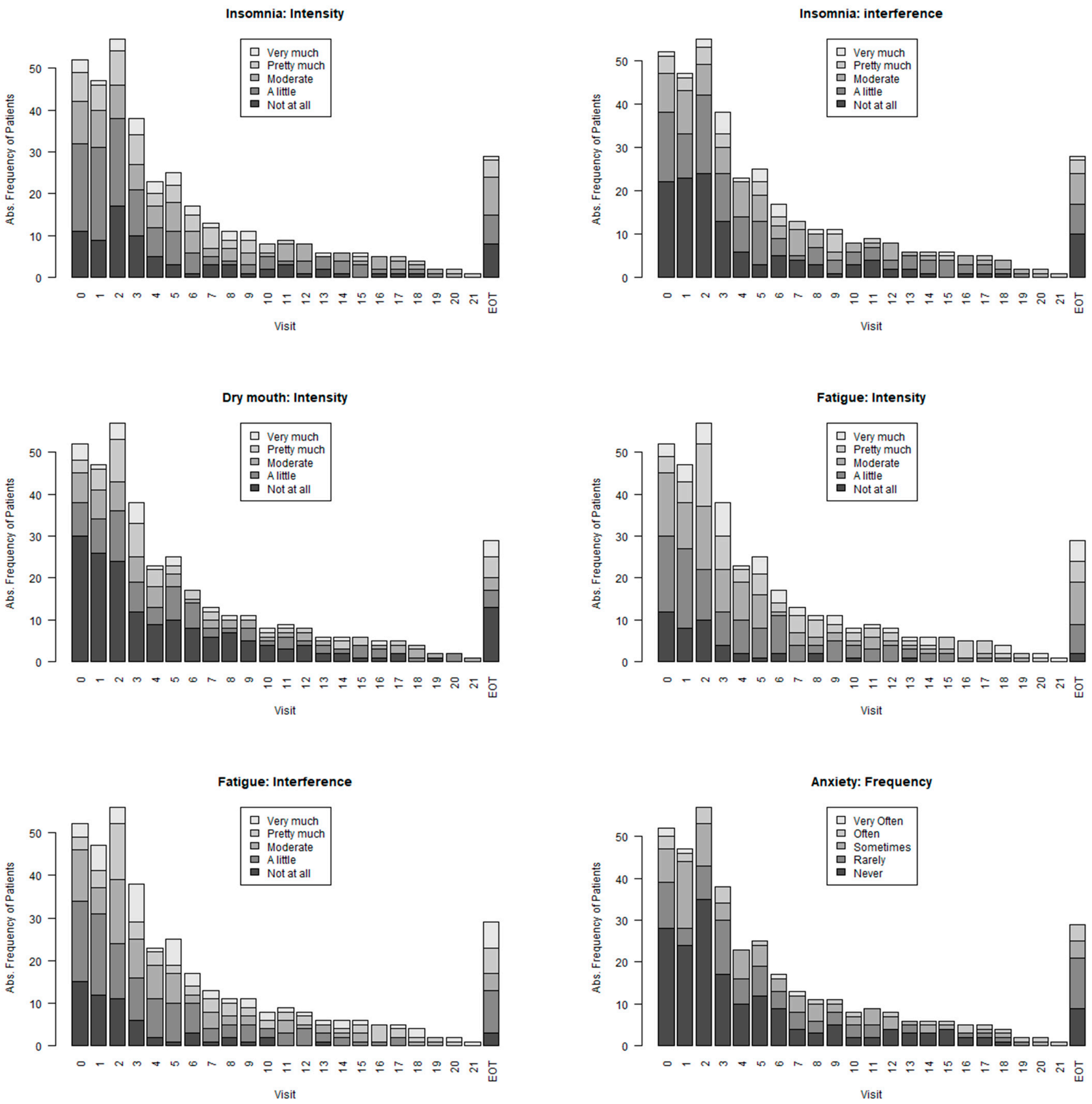
Insomnia: Intensity
Very much
Pretty much
Moderate
A little
Not at all
Abs. Frequency of Patients
Visit
Insomnia: interference
Very much
Pretty much
Moderate
A little
Not at all
Abs. Frequency of Patients
Visit
Dry mouth: Intensity
Very much
Pretty much
Moderate
A little
Not at all
Abs. Frequency of Patients
Visit
Fatigue: Intensity
Very much
Pretty much
Moderate
A little
Not at all
Abs. Frequency of Patients
Visit
Fatigue: Interference
Very much
Pretty much
Moderate
A little
Not at all
Abs. Frequency of Patients
Visit
Anxiety: Frequency
Very Often
Often
Sometimes
Rarely
Never
Abs. Frequency of Patients
Visit

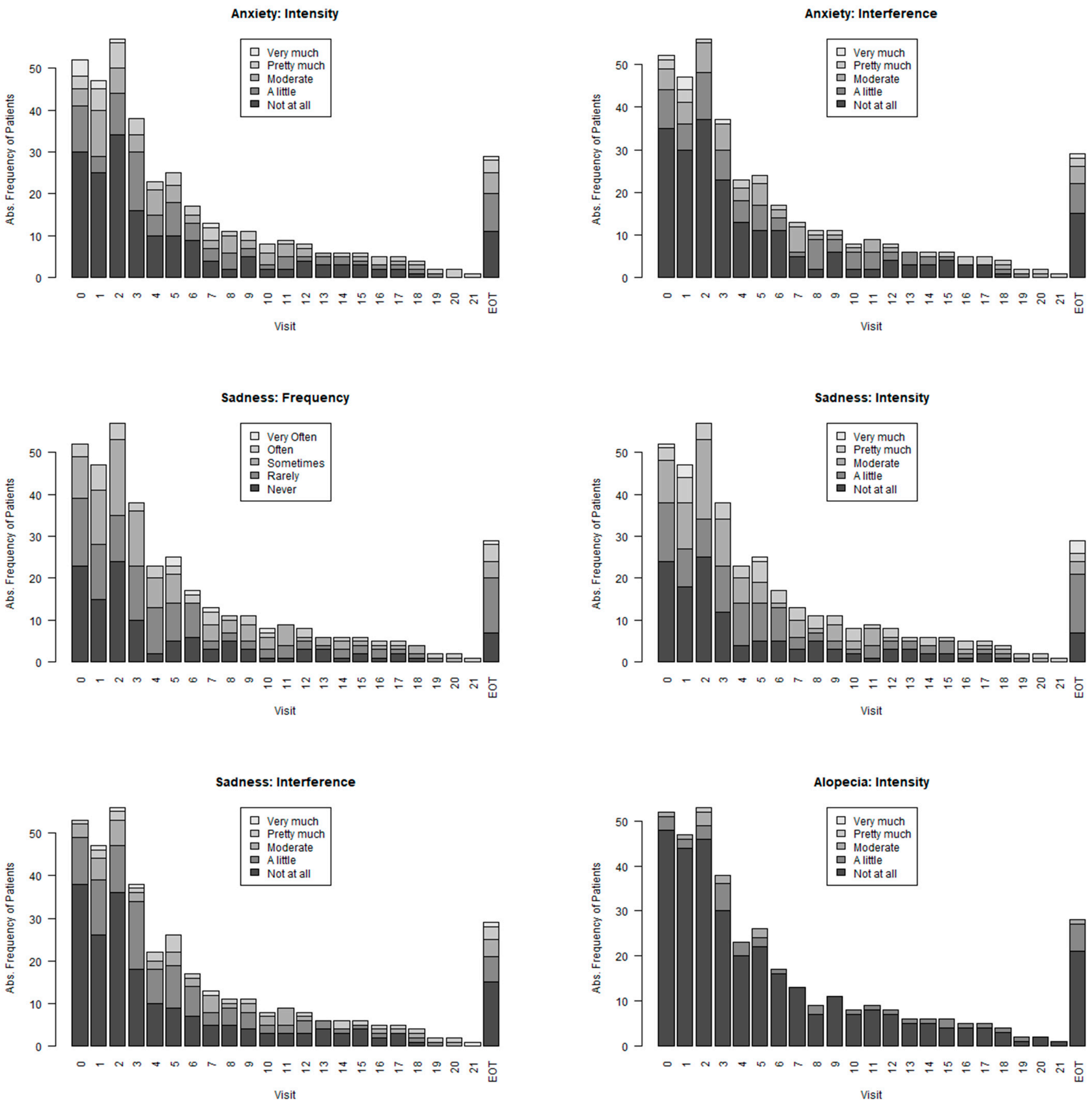
Anxiety: Intensity
Very much
Pretty much
Moderate
A little
Not at all
Abs. Frequency of Patients
Visit
0 1 2 3 4 5 6 7 8 9 10 11 12 13 14 15 16 17 18 19 20 21 EOT
0 10 20 30 40 50
Anxiety: Interference
Very much
Pretty much
Moderate
A little
Not at all
Abs. Frequency of Patients
Visit
Sadness: Frequency
Very Often
Often
Sometimes
Rarely
Never
Abs. Frequency of Patients
Visit
Sadness: Intensity
Very much
Pretty much
Moderate
A little
Not at all
Abs. Frequency of Patients
Visit
Sadness: Interference
Very much
Pretty much
Moderate
A little
Not at all
Abs. Frequency of Patients
Visit
Alopecia: Intensity
Very much
Pretty much
Moderate
A little
Not at all
Abs. Frequency of Patients
Visit

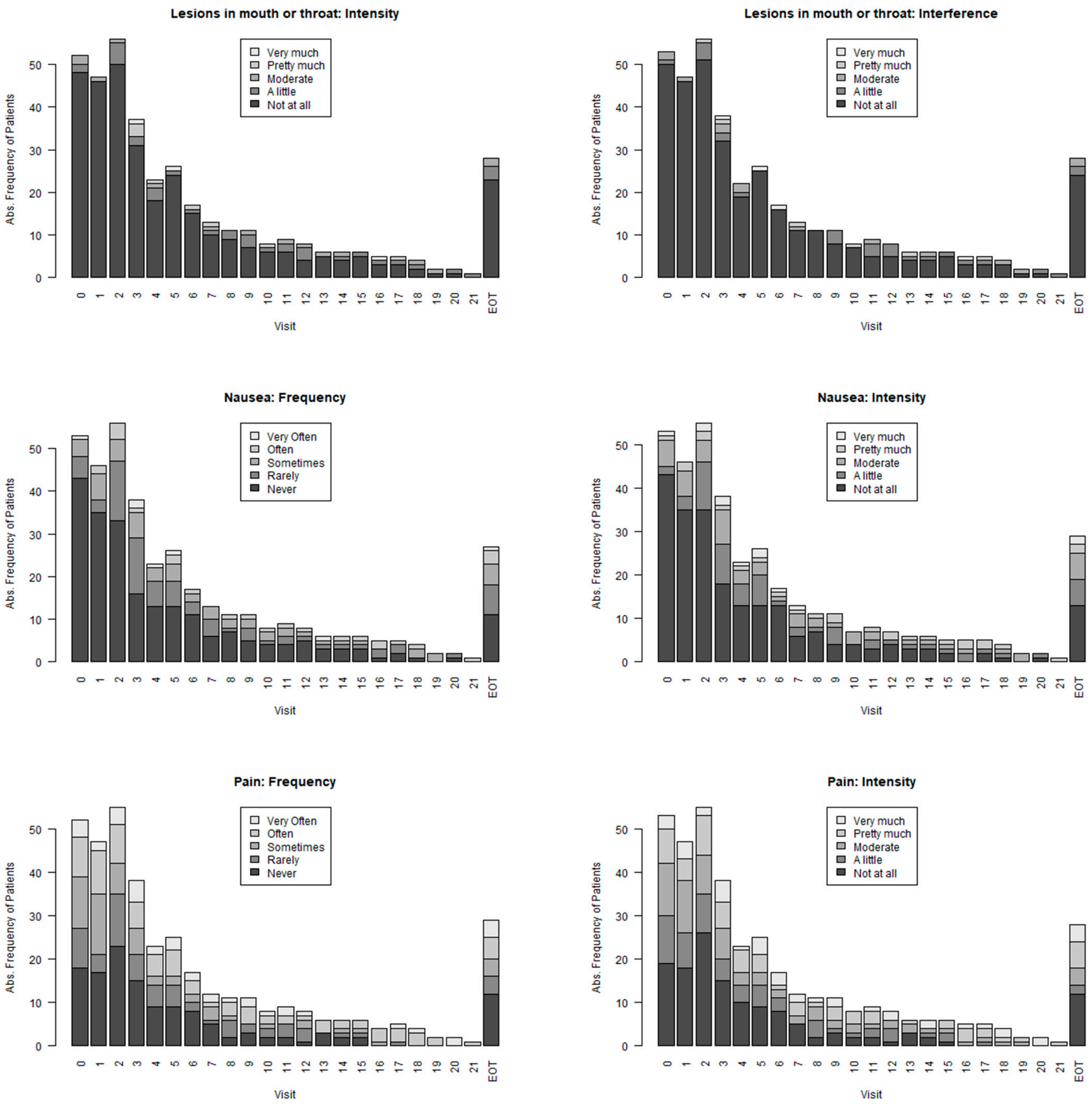
Lesions in mouth or throat: Intensity
Lesions in mouth or throat: Interference
Nausea: Frequency
Nausea: Intensity
Pain: Frequency
Pain: Intensity
Very much
Pretty much
Moderate
A little
Not at all
Very Often
Often
Sometimes
Rarely
Never
Abs. Frequency of Patients
Visit
0 1 2 3 4 5 6 7 8 9 10 11 12 13 14 15 16 17 18 19 20 21 EOT
0 10 20 30 40 50

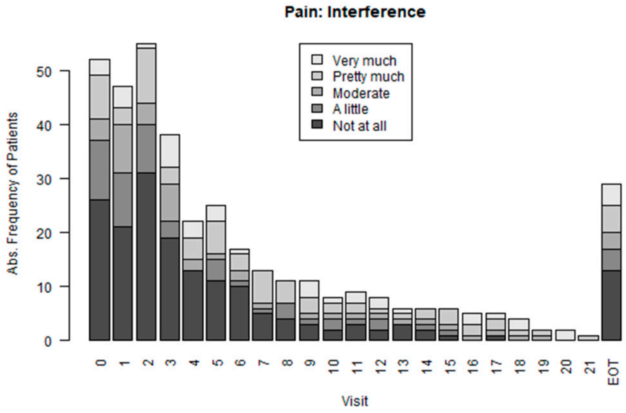
Pain: Interference
Very much
Pretty much
Moderate
A little
Not at all
Abs. Frequency of Patients
Visit
EOT

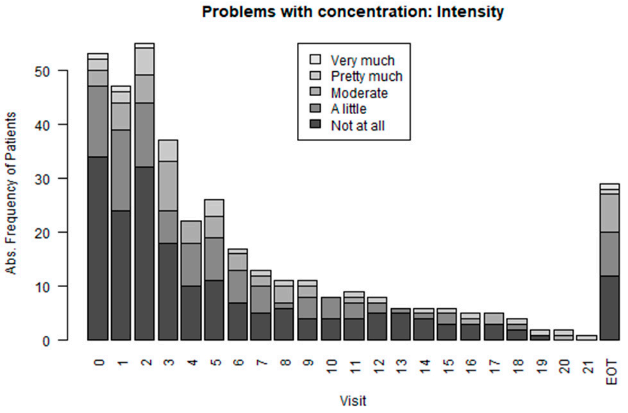
Problems with concentration: Intensity
Very much
Pretty much
Moderate
A little
Not at all
Abs. Frequency of Patients
Visit
EOT

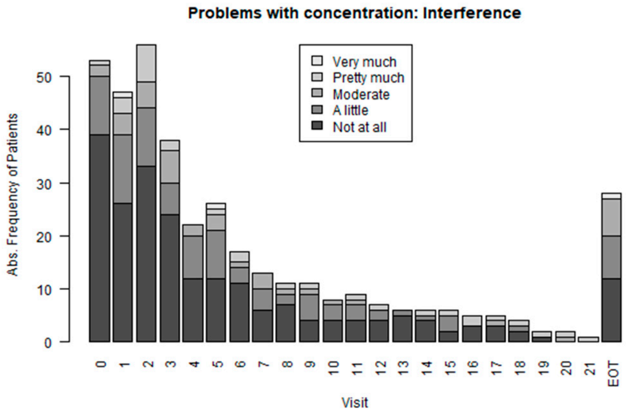
Problems with concentration: Interference
Very much
Pretty much
Moderate
A little
Not at all
Abs. Frequency of Patients
Visit
EOT

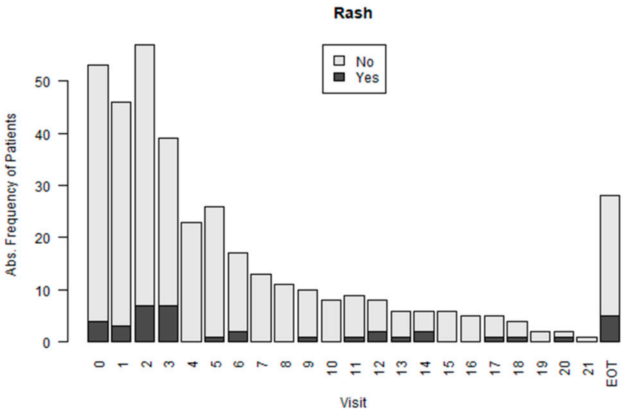
Rash
No
Yes
Abs. Frequency of Patients
Visit
EOT

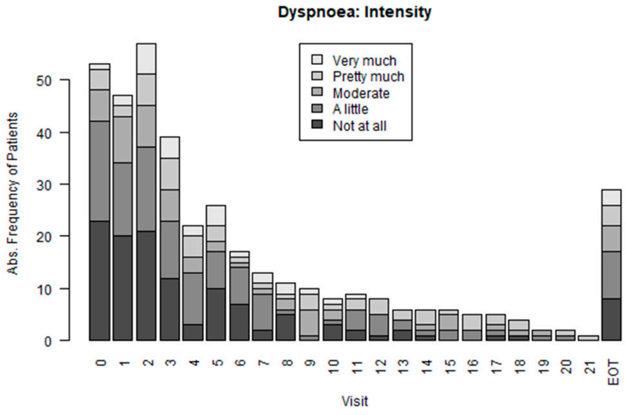
Dyspnoea: Intensity
Very much
Pretty much
Moderate
A little
Not at all
Abs. Frequency of Patients
Visit
EOT

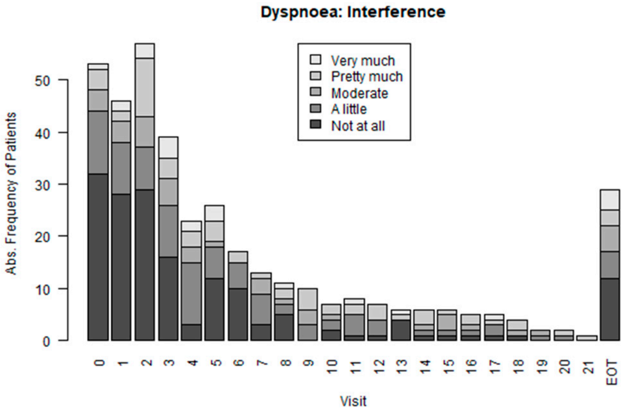
Dyspnoea: Interference
Very much
Pretty much
Moderate
A little
Not at all
Abs. Frequency of Patients
Visit
EOT

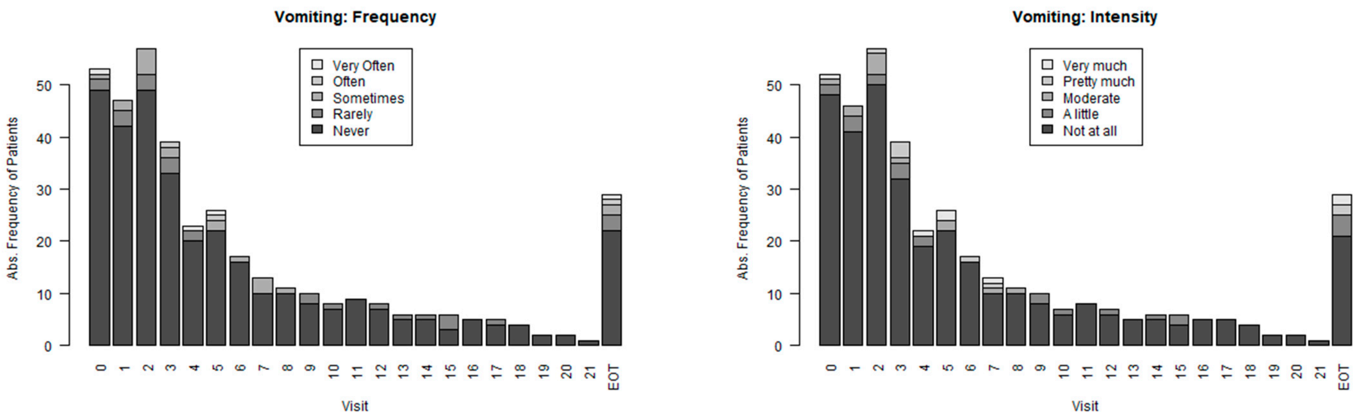

Figure S3. PRO-CTCAE Bar plots by different parameters.
